# Functional and genetic characterization of the non-lysosomal glucosylceramidase 2 as a modifier for Gaucher disease

**DOI:** 10.1186/1750-1172-8-151

**Published:** 2013-09-26

**Authors:** Yildiz Yildiz, Per Hoffmann, Stefan vom Dahl, Bernadette Breiden, Roger Sandhoff, Claus Niederau, Mia Horwitz, Stefan Karlsson, Mirella Filocamo, Deborah Elstein, Michael Beck, Konrad Sandhoff, Eugen Mengel, Maria C Gonzalez, Markus M Nöthen, Ellen Sidransky, Ari Zimran, Manuel Mattheisen

**Affiliations:** 1 Department of Internal Medicine I, University Clinic of Bonn, Bonn, Germany; 2 Department of Internal Medicine, Landeskrankenhaus Bregenz, Bregenz, Austria; 3 Institute of Human Genetics, University Clinic of Bonn, Bonn, Germany; 4 Department of Genomics, Life & Brain Center, University of Bonn, Bonn, Germany; 5 Department of Internal Medicine, St. Franziskus Hospital Köln, Cologne, Germany; 6 Life and Medical Sciences Institute (LIMES) c/o Kekulé-Institute of Chemistry and Biochemistry, University of Bonn, Bonn, Germany; 7 Lipid Pathobiochemistry Group, German Cancer Research Center, Heidelberg, Germany; 8 Department of Instrumental Analytics and Bioanalytics, Technical University for Applied Sciences Mannheim, Mannheim, Germany; 9 Department of Internal Medicine, St. Josef Hospital Oberhausen, Oberhausen, Germany; 10 Department for Cell Research and Immunology, Tel Aviv University, Tel Aviv, Israel; 11 Molecular Medicine and Gene Therapy, Faculty of Medicine, Lund University, Lund, Sweden; 12 Centro di Diagnostica Genetica e Biochimica delle Malattie Metaboliche, G. Gaslini Institute, Genoa, Italy; 13 Shaare Zedek Medical Center, Jerusalem, Israel; 14 Department of Paediatrics, University of Mainz, Mainz, Germany; 15 Section on Molecular Neurogenetics, Medical Genetics Branch, NHGRI, NIH, Bethesda, USA; 16 Department for Genomic Mathematics, University of Bonn, Bonn, Germany; 17 Department of Biomedicine, University of Aarhus, Wilhjelm Meyers Alle 4, 8000, Aarhus C, Denmark

## Abstract

**Background:**

Gaucher disease (GD) is the most common inherited lysosomal storage disorder in humans, caused by mutations in the gene encoding the lysosomal enzyme glucocerebrosidase (*GBA1*). GD is clinically heterogeneous and although the type of *GBA1* mutation plays a role in determining the type of GD, it does not explain the clinical variability seen among patients. Cumulative evidence from recent studies suggests that GBA2 could play a role in the pathogenesis of GD and potentially interacts with GBA1.

**Methods:**

We used a framework of functional and genetic approaches in order to further characterize a potential role of GBA2 in GD. Glucosylceramide (GlcCer) levels in spleen, liver and brain of GBA2-deficient mice and mRNA and protein expression of GBA2 in GBA1-deficient murine fibroblasts were analyzed. Furthermore we crossed GBA2-deficient mice with conditional Gba1 knockout mice in order to quantify the interaction between GBA1 and GBA2. Finally, a genetic approach was used to test whether genetic variation in GBA2 is associated with GD and/ or acts as a modifier in Gaucher patients. We tested 22 SNPs in the *GBA2* and *GBA1* genes in 98 type 1 and 60 type 2/3 Gaucher patients for single- and multi-marker association with GD.

**Results:**

We found a significant accumulation of GlcCer compared to wild-type controls in all three organs studied. In addition, a significant increase of Gba2-protein and Gba2-mRNA levels in GBA1-deficient murine fibroblasts was observed. GlcCer levels in the spleen from Gba1/Gba2 knockout mice were much higher than the sum of the single knockouts, indicating a cross-talk between the two glucosylceramidases and suggesting a partially compensation of the loss of one enzyme by the other. In the genetic approach, no significant association with severity of GD was found for SNPs at the *GBA2* locus. However, in the multi-marker analyses a significant result was detected for p.L444P (*GBA1*) and rs4878628 (*GBA2*), using a model that does not take marginal effects into account.

**Conclusions:**

All together our observations make GBA2 a likely candidate to be involved in GD etiology. Furthermore, they point to GBA2 as a plausible modifier for GBA1 in patients with GD.

## Background

Gaucher disease (GD) is the most common lysosomal storage disease and arises from mutations in the gene encoding the lysosomal glucocerebrosidase (GBA1; EC 3.2.145, MIM# 606463). When GBA1 is absent or impaired, glucosylceramide (GlcCer) accumulates within macrophage lysosomes, leading to liver and spleen enlargement, bone lesions, and in the most severe cases, impairment of central nervous system function [1,2].

Three types of Gaucher disease have been described. Type 1 GD is marked by absence of neurological involvement (non-neuronopathic type) and is the most common form of the disease. It affects approximately 1 in 50,000 individuals [3,4], but is significantly more common among the Ashkenazi Jewish heritage (prevalence up to 1/500 [5]). There is tremendous heterogeneity in the severity of the clinical manifestations of type 1 GD, ranging from patients who are mildly affected to patients who experience life-long debilitating disease. Types 2 and 3 GD are relatively rare and marked by involvement of the central nervous system [6]. While type 2, the acute neuronopathic form of the disease, is characterized by the appearance of several neurologic features, in addition to the severe hepatosplenomegaly, type 3, the subacute neuronopathic form of the disease, is marked by more variable and a less aggressive acceleration of the neurologic manifestations.

More than 330 mutations in the *GBA1* gene have been described to date, by far the most associated with GD [7]. In patients of Ashkenazi Jewish ancestry only six of them account for 90% of disease alleles (c.1226A4G, c.1448T4C, c.84dupG, c.11511G4A, c.1504C4T and c.1604G4A) [8]. The same six mutations account for approx. 50% of disease alleles in non-Jewish patients. Although the type of *GBA1* mutation plays a role in determining the type of Gaucher disease, it does not fully explain the clinical variability seen among patients [9-12]. Therefore, it was hypothesized, that genetic modifiers play a role in the etiology of GD [8].

We and others have previously shown that the enzyme GBA2, besides its known function as hydrolyzing bile acid 3-O-glucosides in the liver as endogenous compounds [13,14], also hydrolyzes glucosylceramide [15]. In accordance with this, GBA2-deficient mice show an accumulation of GlcCer in different tissues [15]. Moreover, a crosstalk of GBA1 and GBA2 in the metabolism of glycosphingolipids has recently been hypothesized [16] and a subsequent study suggested a particular metabolic role of GBA2 in the brain [17].

In the present study, we explored whether the non-lysosomal glucocerebrosidase (GBA2) could play a role as modifier for Gaucher disease. We examined the potential role of *GBA2* as a modifier of Gaucher disease and the crosstalk between GBA1 and GBA2 using three subsequent steps. In a first step, we aimed to further explore the biochemical characteristics of GBA2-deficient mice. Therefore, we analyzed GlcCer levels in spleen, liver and brain of GBA2-deficient mice, since these are the predominantly affected organs in GD. In a second step we aimed to further characterize the potential interaction between GBA1 and GBA2. We investigated whether GBA2 expression is altered in fibroblasts of GBA1-deficient mice to obtain further evidence for an interaction between lysosomal and non-lysosomal glycosylases. Finally, we crossed our GBA2-deficient mice with conditional GBA1-knockout mice [18] in order to quantify the interaction between GBA1 and GBA2. Since the results in the functional steps highly supported such an interaction we used, in a third step, a genetic approach to directly test whether genetic variation in *GBA2* acts as a modifier in Gaucher patients.

## Methods

### Lipid analysis

Spleen, liver and brain was homogenized, lyophilised and extracted as describe previously [19]. Protein and cell debris were separated by filtration. The phospholipids were degraded by mild alkaline hydrolysis with 50 mM sodium hydroxide in chloroform/methanol (1:1 (v/v)). After neutralization with glacial acetic acid, sphingolipids were desalted by reversed-phase chromatography, separated into acidic and neutral glycosphingolipids by anion exchange chromatography with DEAE-cellulose [20].

For separation of polar neutral lipids by thin layer chromatography (TLC), samples were applied to prewashed (chloroform/methanol 1:1 (v/v)) thin layer Silica Gel 60 plates (Merck, Darmstadt, Germany) and the chromatograms were developed with chloroform/methanol/water (70/30/5, v/v/v). Hexosylceramide (HexCer) were separated into GlcCer and galactosylceramide on borate-impregnated TLC plates [21] developed in chloroform/methanol/water (65/25/4, v/v/v). After development, plates were air-dried, sprayed with 8% (w/v) H_3_PO_4_ containing 10% (w/v) copper (II) sulfate pentahydrate, and charred for 10 min at 180°C, and lipids were quantitated by photo densitometry (Camac, Muttenz, Switzerland) at λ = 595 nm.

For mass spectrometric analysis, aliquots of the neutral lipid extracts were mixed with an appropriate amount of internal standards containing the GlcCer-species: GlcCer(d18:1;14:0), GlcCer(d18:1;19:0), GlcCer(d18:1;25:0), and GlcCer(d18:1;31:0). Tandem Mass spectrometric analysis was performed using a triple quadrupole instrument (VG Micromass, Cheshire, UK) equipped with a nano-electrospray source and gold-sputtered capillaries. Parameters for cone voltage and the collision energy of the different scan modes used, and sphingolipid quantification was performed as previously described [22,23].

### GBA2 expression in fibroblasts of GBA1-deficient mice

#### Preparation of cultured fibroblasts and western blot analyses

Embryonic murine primary fibroblasts were generated from the GBA1-deficient mice [24] and cultured to early confluency and harvested as described earlier [25]. Total fibroblasts extracts were prepared as described previously [25]. Equal amounts of protein (20–40 μg/lane) were separated by sodium dodecyl sulfate-polyacrylamide gel electrophoresis (SDS-PAGE), transferred to nitrocellulose and incubated with anti-GBA2 [15], followed by anti-rabbit secondary antibody (Calbiochem, La Jolla, CA, USA). To confirm equal loading, blots were re-probed with anti-β-actin primary antibody (Sigma-Aldrich Chemie GmbH, Taufkirchen, DE). Immunoreactive proteins were detected using the ECL system (Amersham Biosciences, Piscataway, NJ, USA).

#### Reverse transcriptase-PCR (RT-PCR)

RNA isolation, reverse transcription, and RT-PCR were performed as previously described [15]. RNA isolated from fresh or shock-frozen fibroblasts with Trizol (Invitrogen, Karlsruhe, DE) according to the manufacturer's guidelines. For each sample, 1 μg total RNA was used. Before reverse transcription, samples were DNA-digested by incubation with RQ1 RNase-free DNAse (Promega, Madison, WI, USA). Reverse transcription was performed using reverse transcriptase (Invitrogen, Karlsruhe, DE) and random primers (Microsynth, Balgach, CH). Primers and probes (mM00554547_m1GBA2, mM00484700_m1GBA1,) for detection of targets and house-keeping gene (18SrRNA) were provided by Applied Biosystems (Foster City, USA) as ready-to-use mixes and used according to the manufacturer's guidelines. RT-PCR was performed using the ABI 7700 sequence detector (Applied Biosystems, Life Technologies Corporation, Carlsbad, CA, USA).

### Glycolipids accumulation in GBA1- and GBA2- deficient mice

#### Generation of mice and characterization of glycolipids accumulation

The conditional *Gba-* knockout mice were kindly provided by Stefan Karlsson, University of Lund, Sweden [18]. We crossed those mice to our GBA2-deficient mice and deleted *Gba1* specifically in the liver and spleen by Cre-dependent recombination. All mice received a series of five polyinosinic–polycytidylic acid injections starting within the first week of life to induce excision of “floxed” exons. The pups tolerated the treatment well. Complete excision of *GBA1* exons 9–11 in liver, and spleen was confirmed by different PCR analysis as described previously [18].

#### Statistical analyses

In order to assess the significance of accumulation of GlcCer in the different mutant and control mice we used a paired Student’s t tests for pair-wise comparisons, data are expressed as mean ± standard deviation. Significance was tested at the level of P < 0.05.

### Genetic variation in *GBA2* as a modifier in Gaucher patients

#### DNA extraction, SNP selection, and genotyping

Informed consent was provided under an Institute Review Board approved clinical protocol to analyse the *GBA1* and *GBA2* gene locus in Gaucher patients. Ethylenediaminetetraacetic acid anti-coagulated venous blood samples were collected from all participating individuals. Lymphocyte DNA was isolated by salting- out [8] with saturated sodium chloride solution or by a Chemagic Magnetic Separation Module I (Chemagen, Baesweiler, DE) used according to the manufacturer’s recommendations.

*GBA2* is located on chromosomes 9p13.3 and spans around 13.1 kb. For our finemapping approach we used Haploview V4.1 [26] to select all SNPs with a maximum r^2^ value of 0.8 (pairwise tagging approach) and a minor allele frequency (MAF) of at least 10% in CEU HapMap individuals [24]. In addition, p.N370S and p.L444P, the first ever described and still predominant mutations in *GBA1* around the globe were genotyped. While homozygosity for p.N370S is usually associated with the non-neuronopathic type (type 1 GD), the same is true for p.L444P and a neuropathic phenotype (types 2 and 3 GD). Hence, these two mutations are perfectly suitable to test if variants at the *GBA2* (selected by a haplotype tagging approach, see above) locus have a modifying effect on the association of mutations in *GBA1* with severity of GD.

Sequences were retrieved from CHIP Bioinformatics Tools (http://snpper.chip.org/). We genotyped 98 DNA samples from type 1 and 60 DNA samples from type 2/3 Gaucher patients, all of European ancestry, and provide by different clinical centres throughout Europe, specialized in treatment and research of Gaucher disease. The selection of the aforementioned patient groups with type 1 and type 2/3 GD follows the assumption that type 1 in general depicts a milder form and type 2/3 a more severe form of GD. In addition, a more important role for GBA2 in the brain has been described previously [17], making an involvement of GBA2 in the etiology of type 2/3 GD more likely. Hence, the results of subsequent testing for allele frequency differences in these groups can be interpreted to be related to severity in GD. A total of 26 SNPs (24 for *GBA2* and 2 for *GBA1*) were included in the assay. Genotyping was performed on genomic DNA using the Sequenom MALDI-TOF mass-spectrometer (Sequenom iPlex assay) and were analyzed using the Spectrodesigner Software package (Sequenom, San Diego, CA, USA). Primers were synthesized at Metabion, Germany. All primer sequences are available upon request. Only SNPs forming three distinct clusters in the Sequenom Typer Analysis software were included in the analysis.

#### Statistical analysis

In the association analysis for genetic variation in *GBA1* and *GBA2* all quality control (QC) steps and single-marker analyses were performed using PLINK [27]. The subsequent multi-marker analyses were performed using INTERSNP [28]. As a first step we analyzed p.N370S and p.L444P (*GBA1*), as well as the variants at the *GBA2* gene locus with respect to their association with the severity of Gaucher disease (as defined above). In order to test if variants at the *GBA2* locus have a modifying effect on the association of p.N370S and/ or p.L444P with severity of GD, we also conducted multi-marker analyses aiming to identify epistatic effects. We therefore performed in a subsequent step an interaction analyses using a logistic regression framework and testing for an additional allelic (2 degrees of freedom (d.f.)) or genotypic effect (6 d.f.) of SNPs at the *GBA2* locus on the aforementioned association of p.N370S or p.L444P. In addition, we used a log-linear model (4 d.f.) in order to test for an epistatic effect of SNPs at the *GBA2* locus and p.N370S or p.L444P. This analysis was performed without taking marginal effects at both gene loci into account.

## Results

### Increased GlcCer level in GBA2-deficient mice liver, spleen and brain

**Figure 1:**
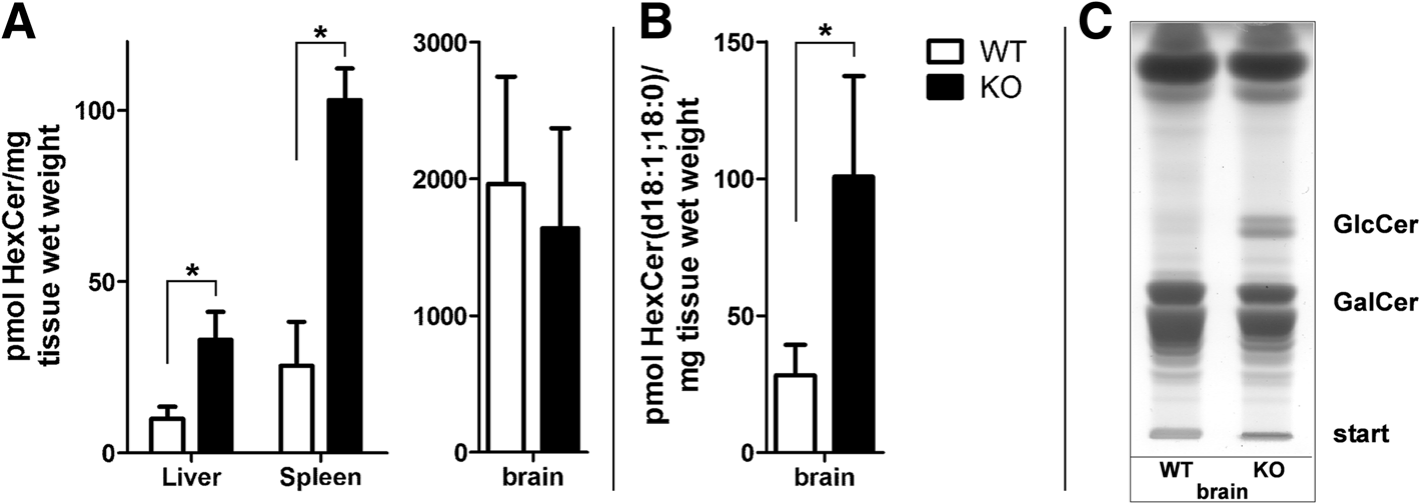
**Quantitative mass spectrometric analysis of HexCer (sum of GlcCer and GalCer). (A)** Total HexCer amount in liver, spleen, and brain of 6 -month-old GBA2-deficient (KO) and wild-type (WT) mice. **(B)** Amount of the HexCer species carrying the typical neuronal ceramide anchor with a stearic acyl residue. *: P < 0.05, n = 4 animals per group. **(C)** TLC of a representative brain lipid sample in which GlcCer and GalCer are separated. Note the increased GlcCer in the KO. The double band reflects heterogeneity of its ceramide anchor composition.

Although GBA2-deficient mice do not show any hepatosplenomegaly, the quantitative analysis of HexCer amount in liver, spleen and brain shows a significant accumulation of HexCer by mass spectrometry in liver and spleen and a significant increase of those HexCer species being likely of neuronal origin in brain. As liver and spleen do not contain substantial amounts of (GalCer),the measured HexCer basically represents GlcCer. The brain however is full of galactosylceramide. Therefore, GlcCer and GalCer (in sum HexCer) of brain samples were separated on TLC demonstrating the increase of GlcCer (Figure 1). These results underline the important role of GBA2 in the homeostasis of GlcCer in these organs.

### Increased mRNA and protein expression of GBA2 in GBA1-deficient mice fibroblasts

**Figure 2:**
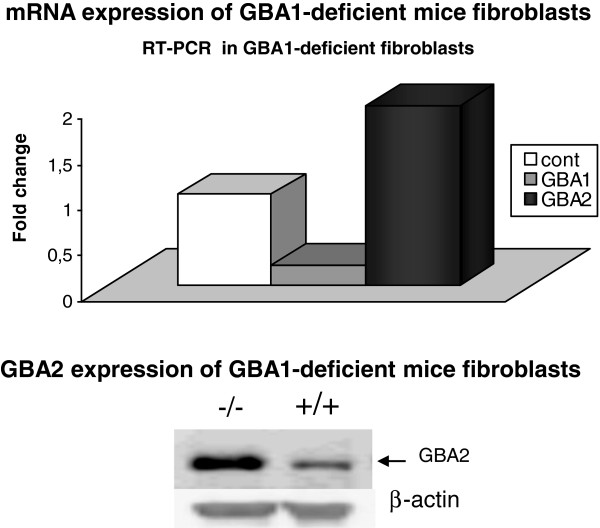
**mRNA expression of *GBA2 *and *GBA1 *in GBA1-deficient embryonic mice fibroblasts **[24]**expressed as fold change *GBA2 *versus *GBA1***. 18S-RNA was used as internal control. Western blot analysis for GBA2 in GBA1-deficient embryonic mice fibroblasts, beta-actin was used as loading control.

As shown in Figure 2, we observed a clear decrease of GBA1 protein level and mRNA expression in GBA1-deficient fibroblasts. Furthermore, we observed a strong increase of GBA2 protein and mRNA expression, indicating that GBA2 is up regulated, following a compensatory mechanism, that is aimed to adjust for the lack of GBA1.

### Glycolipids accumulation in GBA1 and GBA2 deficient mice

**Figure 3:**
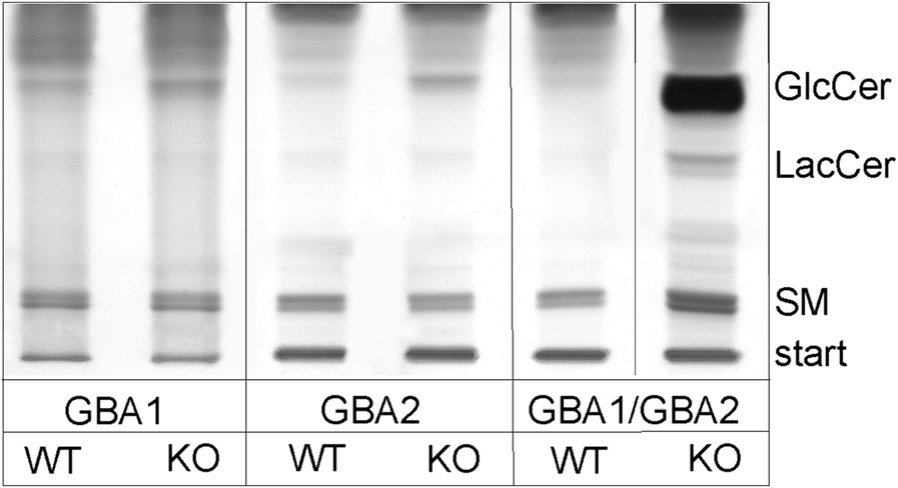
**GlcCer levels in GBA-deficient mice.** Thin layer chromatography (TLC) analysing glycosphingolipids from spleen of 12-month-old GBA1-deficient, GBA2-deficient, and GBA1/GBA2-deficient mice. Representative TLC analysis shown neutral sphingolipids of 5 mg (wet weight). WT: wild-type, KO: knockout mice, GlcCer: glucosylceramide, LacCer: lactosylceramide, SM: sphingomyelin.

We analysed GlcCer levels in the spleen from mice that were deficient for either GBA1 or GBA2 or both. GlcCer accumulated in the spleen from all three knockout mice. However, GlcCer levels in the spleen from *Gba1*/*Gba2* knockout mice were much higher than the sum of the single knockouts (Figure 3). Our results indicate that there is a cross-talk between the two glucosylceramidases and suggest that one enzyme can partially compensate the loss of the other.

### Single- and multi-marker analysis for *GBA2* and *GBA1* in Gaucher patients

From the initially selected 24 SNPs for *GBA2* and 2 mutations in *GBA1* (p.N370S and p.L444P), a total of 19 (17 for *GBA2* and 2 for *GBA1*) variants performed well during genotyping and passed standard quality control (QC) procedures. Out of seven SNPs that were not put forward to statistical analysis, 1 SNP was dropped due to low call rate and an additional 6 SNPs failed tests for differences in missingness patterns between patients with type 1 and type 2/3 GD, deviation from Hardy Weinberg Equilibrium (< 0.001) or a minimum minor allele frequency of at least 1% in cases and controls. Samples were excluded from the analysis in case they were not successfully genotyped for > 3 SNPs in the post-SNP-QC datasets. A total of 86 patients with type 1 and 48 patients with type 2/3 GD could be incorporated into the analyses. In the single-marker analyses we observed (as expected) an association of p.N370S and p.L444P with the severity of Gaucher disease in the single-marker analysis (N370S: OR = 0.0047, CI [0.001/0.023], *P*_*single*_ *=* 8.22 × 10^-11^; L444P: OR = 4.54, CI [2.41/8.54], *P*_*single*_ = 2.69 × 10^-6^). In contrast, we did not observe a significant association with severity of GD in single-marker analyses for SNPs at the *GBA2* locus (the best result was obtained for rs10972579: OR = 2.192, CI [0.82/5.86], *P*_*single*_ = 0.118). In the multi-marker analysis, no additional effect (2 and 6 d.f. model) was observed for SNPs at the *GBA2* locus on the association of p.N370S / p.L444P with severity of GD (Table 1). In addition, no epistatic effect (4 d.f. model) was observed when p.N370S was one of the two SNPs in the analysis (Table 2). In contrast, a significant result was observed in case p.L444P was paired with SNPs at the *GBA2* locus. The significant combination included rs4878628 at the *GBA2* locus and reached a *P*_*corr*_ = 0.027. It is of note, that rs4878628 itself, other than p.L444P, has no marginal effect on its own (OR = 0.999, CI [0.54/1.85], *P*_*single*_ = 0.998, Table 2).

**Table 1:** Tests for additional allelic or genotypic effect on association of p.L444P and p.N370S with severity of Gaucher disease

						**ALLELIC**		**GENOTYPIC**	
***GBA2 *SNP**	**POS**_**hg18**_	**MA**	**MAF**_**co**_	**MAF**_**ca**_	***P***_***single***_	***P***_***L444P***_	***P***_***N370S***_	***P***_***L444P***_	***P***_***N370S***_
rs10814274	35724942	T	0.494	0.479	0.811	0.3971	0.1444	0.0585	0.3160
rs34312177	35730649	T	0.067	0.057	0.769	**0.0213**	0.3679	0.0650	0.5723
rs3833700	35738696	T	0.302	0.292	0.847	0.3050	0.4095	**0.0197**	0.7977
rs1570246	35738843	A	0.494	0.479	0.811	0.3971	0.1444	0.0585	0.3160
rs3750434	35738985	T	0.500	0.479	0.739	0.4098	0.1445	0.0620	0.3213
rs1570247	35739264	T	0.500	0.500	1.000	0.4339	0.3288	0.0735	0.4953
rs2236288	35739837	G	0.186	0.219	0.518	0.0895	0.9200	0.1329	0.7770
rs1570249	35741250	T	0.494	0.479	0.811	0.3971	0.1444	0.0585	0.3160
rs2145923	35742243	C	0.157	0.125	0.480	0.6267	0.2096	0.3936	0.3599
rs1322045	35742487	C	0.300	0.292	0.880	0.3138	0.4113	**0.0205**	0.7973
rs1570250	35742683	T	0.302	0.292	0.847	0.3050	0.4095	**0.0197**	0.7977
rs34478611	35743925	T	0.204	0.229	0.622	0.8899	0.6611	0.1400	0.4579
rs4878628	35744491	T	0.234	0.234	0.998	0.2716	0.6676	**0.0058**	0.7601
rs10814275	35748564	G	0.155	0.117	0.408	0.1883	0.4275	0.3808	0.7907
rs1570248	35756549	G	0.302	0.292	0.847	0.3050	0.4095	**0.0197**	0.7977
rs10972579	35766001	T	0.055	0.106	0.118	0.1054	0.6855	**0.0344**	0.8601
rs10972581	35769559	T	0.471	0.479	0.900	0.9124	0.2853	0.2255	0.3993

*MA* Minor Allele, *MAF*_*co*_ frequency of MA in patients with type 1 GD, *MAF*_*ca*_ frequency of MA in patients with type 2/3 GD, *P*_*single*_ p-value for logistic regression single-marker analysis, *P*_*L444P*_ and *P*_*N370S*_ P-values (uncorrected) for additional allelic and genotypic effect of *GBA2* SNP on p.L444P and p.N370S association, respectively. P-values are in bold in case they reach the level of nominal significance (*P* < 0.05).

**Table 2:** Tests for epistatic effect of p.L444P or p.N370S and markers at GBA2 locus on severity of Gaucher disease

				**p.L444P**		**p.N370S**	
***GBA2 *SNP**	**POS**_**hg18**_	**MA**	***P***_***single***_	***P***_***nom***_	***P***_***corr***_	***P***_***nom***_	***P***_***corr***_
rs10814274	35724942	T	0.811	**0.0224**	0.3195	0.4648	1.0000
rs34312177	35730649	T	0.769	0.0672	0.6933	0.4809	1.0000
rs3833700	35738696	T	0.847	**0.0045**	0.0735	0.9861	1.0000
rs1570246	35738843	A	0.811	**0.0223**	0.3195	0.4648	1.0000
rs3750434	35738985	T	0.739	**0.0235**	0.3328	0.4673	1.0000
rs1570247	35739264	T	1.000	**0.0253**	0.3528	0.8580	1.0000
rs2236288	35739837	G	0.518	0.1145	0.8734	0.9957	1.0000
rs1570249	35741250	T	0.811	**0.0224**	0.3195	0.4648	1.0000
rs2145923	35742243	C	0.480	0.4546	1.0000	0.8296	1.0000
rs1322045	35742487	C	0.880	**0.0048**	0.0787	0.9844	1.0000
rs1570250	35742683	T	0.847	**0.0045**	0.0735	0.9861	1.0000
rs34478611	35743925	T	0.622	**0.0428**	0.5245	0.9975	1.0000
rs4878628	35744491	T	0.998	**0.0016**	**0.0272**	0.9886	1.0000
rs10814275	35748564	G	0.408	0.6472	1.0000	0.6777	1.0000
rs1570248	35756549	G	0.847	**0.0045**	0.0735	0.9861	1.0000
rs10972579	35766001	T	0.118	0.0548	0.6163	0.7097	1.0000
rs10972581	35769559	T	0.900	0.0995	0.8316	0.2835	0.9965

*MA* Minor Allele, *P*_*single*_ p-value for logistic regression single-marker analysis, *P*_*nom*_ and *P*_*corr*_ p-Values for genotypic interaction without taking marginal effects into account (nominal and corrected for number of markers at *GBA2* locus). P-values are in bold in case they reach the level of nominal significance (*P* < 0.05).

## Discussion

In patients with Gaucher disease, hepatosplenomegaly due to accumulation of GlcCer particularly in cells of the macrophage lineage in liver and spleen is one characteristic symptom. For all three organs (spleen, liver, and brain) we were able to show that their GlcCer levels were significantly elevated in GBA2-deficient mice. Hence, a pattern of GlcCer accumulation was observed, that is potentially relevant to GD. It is of note, that to date it is not clear how GlcCer itself or the consequent imbalances of ceramide, sphingosine, and sphingosine 1-phosphate affects Gaucher disease. Furthermore, it is unknown how GlcCer accumulation in lysosomes leads to cellular pathology, and whether GlcCer can escape the lysosomes and interact with different cellular and biochemical pathways in other organelles [29]. Different studies implicate profound systematic pathophysiological changes rather than simple lipid accumulation as the basis of GD. Previous studies on biochemical and pathological analyses demonstrated a relationship between the amount of tissue glucosylceramides and different gene expression profile alterations [30]. Further it was shown that increases and decreases in glucosylceramide levels can dramatically alter the endocytic targeting of lactosylceramide and suggested a role for glucosylceramide in regulation of membrane transport [31].

Encouraged by our observation of significantly elevated GlcCer levels in GBA2-deficient mice we aimed to further characterized a potential interaction of GBA1 and GBA2 and shed light on the GlcCer pathophysiology in GD. Our observation that the GBA2 mRNA and protein expression is clearly increased in fibroblasts of GBA1-deficient mice indicates that GBA2, as a compensatory mechanism aimed to adjust for the lack of GBA1, is up-regulated. In addition, simultaneous absence of GBA1 and GBA2 function seems to have a higher impact on accumulation of GlcCer in GD relevant organs than loss of function respectively in GBA1 and GBA2 alone.

Using a high-resolution strategy we disappointingly found none of the common variants at the *GBA2* locus to be associated with severity. We detected, however, an epistatic effect on the severity of Gaucher disease for p.L444P and rs4878628 (*P*_*corr*_ = 0.027). It is of note, that this result is purely based on the interaction term and does not take into account the marginal effects (namely for p.L444P) at the two gene loci. No epistatic effect was detected for p.N370S and common variants at the *GBA2* gene locus. This observation comes in accordance with the fact that homozygosity for p.L444P (and not p.N370S) is usually associated with a neuropathic phenotype and that GBA2 has recently been identified to mainly play a role in the brain [17]. Although the detection of an epistatic effect of variants at the *GBA1* (p.L444P) and *GBA2* (rs4878628) loci is an encouraging result in terms of our initial hypothesis, it is reasonable to assume that our study did not have enough power to detect some of the possible modifier variants at the *GBA2* locus, both, at the single- as well as on the multi-marker level. It is of note, that due to an already limited sample size, we did not build separate samples of patients with type 2 and type 3 GD to allow for an I depth characterization of our association finding or to find any other association signal at the GBA2 gene locus. In addition, using a haplotype tagging approach for SNPs at the *GBA2* gene locus (with a MAF > 10%) might have led to an oversight for signals from rare (and potentially functional relevant) variants in the region. However, the size of our sample did make such an observation a priori unlikely and thus we focus our study on identification of common variants.

## Conclusion

Our functional results all together provide further evidence for the involvement of GBA2 as a likely candidate in the etiology of Gaucher disease. Furthermore, they point to GBA2 as a plausible modifier for GBA1 in patients with GD. Beyond that, our results in the genetic approach, i.e. the identification of an epistatic effect involving p.L444P and a marker at the *GBA2* locus, point to a potential role of GBA2 as a modifier mainly for type 2 and 3 Gaucher disease. Limitations due the overall size of our genetic study, however, make it difficult to rule out a potential role also for type 1 Gaucher disease. Independent studies are warranted to follow up on our interaction finding and to further investigate the role of GBA2 in the clinical expression of Gaucher disease. Furthermore, more detailed studies of GBA2 and the *Gba2* knockout mice are warranted to provide additional information about maintaining the homeostasis of glucosylceramide, ceramide, and other sphingolipids concentrations in different cell types.

## Competing interests

The authors declared that they have no competing interests.

## Authors’ contributions

YY, PH, MMN, and MM designed the study, interpreted the results and wrote the manuscript with feedback from the other authors. SvD, CN, MF, MB, EM, MH, DE, ES, and AZ recruited and diagnosed the Gaucher patients and provided the genotypes for the genetics approach. BB and KS performed the thin layer chromatography; RS the mass spectrometric analysis, MCG the western blot analyses; SK provided the Gba1 ko-mice and performed PCR for GBA1. YY in addition engineered the Gba1/Gba2 double ko-mice, and performed the RT-PCR experiment; MM and PH in addition performed the statistical analyses for the genetics approach. All authors read and approved the final manuscript.
